# Using road traffic accidents data for hotspots identification: a case study in Marand, Iran

**Published:** 2019-08

**Authors:** Ebrahim Khalil Pour, Kiomars Mohaddes, Mohammad Saadati

**Affiliations:** ^*a*^Traffic Police, Tabriz, East Azerbaijan Province, Iran.; ^*b*^Road Traffic Injury Research Center, Tabriz University of Medical Sciences, Tabriz, Iran.

## Abstract

**Background:**

Identification of the hotspots is the first step to for safety promotion. Road traffic accidents data is the best source to know the hotspots and draw the future interventions. The aim of this study was to identify the Marand city hotspots based on road traffic accidents data during 2015-2018.

**Methods:**

This was a cross-sectional study conducted in 2019. Traffic Police database was used to extract the road traffic accidents data. Accident type, cause, severity (death, injured and etc), location, month and year were included in the extracted data. P indicator (including all kinds of the accidents severity/ time and road type variables) was estimated for each of the identified zones. Field visit was done to identify the safety defects. Data were analyzed using Excel 2016.

**Results:**

Totally 1251 traffic accidents were occurred during 3 years in Marand city, which had led to 26 deaths. 28.8% and 56.83% of the accidents were related to the pedestrians and motorcycles, respectively. Distracted driving and non-respect of priority road were the main reason of the accidents. Based on the P indicator, Emam Hossein Square (p=36.99), Yaldir Bridge zone (p=35.6) and Abasi Street (p=35.6) were the most dangerous zones in Marand city. Safety promotion suggestions were provided for each of the hotspots based on the field visit.

**Conclusions:**

Accidents data provides us a vision to identify the hotspots and they could be used for evidence-based safety promotion. Safety promotion initiatives must be implemented through intersect-oral collaboration based on identified priorities.

**Keywords:**

Road traffic accidents, Hotspots, Safety promotion

